# Substitution

**Published:** 1887-03

**Authors:** 


SUBSTITUTION.—Does the profession realize how much injury is done to physicians and their patients by the substitution of spurious, or the so-called "just as good" preparations, in place of goods of standard reputation? The following letter from Dr. Springer is a case in point.

Respectfully,

BATTLE & CO.,

*Chemists Corporation.*

VAN BUREN, O., Sept. 10, 1886.

*Messrs. Battle & Co., St. Louis, Mo.:*

GENTLEMEN—In the case of "insomnia," which I reported to you in May last, and wherein it required seven drachm doses (hourly, one drachm,) to produce sleep by Bromidia bought at pharmacy in Findlay, it required but one drachm, repeated in one hour, to produce a good night's rest, of the sample bottle you sent me. I also use the Bromidia (Battle & Co.) with the best results in "cholera infantum" and in "hysteria." *Am satisfied that the article bought at Findlay was "spurious."*

GEORGE SPRINGER, M. D.

